# Detection of a microbial source tracking marker by isothermal helicase-dependent amplification and a nucleic acid lateral-flow strip test

**DOI:** 10.1038/s41598-018-36749-7

**Published:** 2019-01-23

**Authors:** Claudia Kolm, Roland Martzy, Manuela Führer, Robert L. Mach, Rudolf Krska, Sabine Baumgartner, Andreas H. Farnleitner, Georg H. Reischer

**Affiliations:** 1 TU Wien, Institute of Chemical, Environmental & Bioscience Engineering, Molecular Diagnostics Group, Department IFA-Tulln, Tulln, Austria; 2 ICC Interuniversity Cooperation Centre Water & Health, Vienna, Austria; 3 University of Natural Resources and Life Sciences, Vienna (BOKU), Department IFA-Tulln, Center for Analytical Chemistry, Tulln, Austria; 4 TU Wien, Institute of Chemical, Environmental & Bioscience Engineering, Research Division Biochemical Technology, Research Group Synthetic Biology and Molecular Biotechnology, Vienna, Austria; 5 Queen’s University Belfast, School of Biological Sciences, Institute for Global Food Security, Belfast, Northern Ireland, United Kingdom; 6 Karl Landsteiner University of Health Sciences, Research Unit Water Quality and Health, Krems, Austria; 7 TU Wien, Institute of Chemical, Environmental & Bioscience Engineering, Research Division Biochemical Technology, Research Group of Environmental Microbiology and Molecular Diagnostics, Vienna, Austria

## Abstract

Over the last decades, various PCR-based methods have been proposed that can identify sources of faecal pollution in environmental waters. These microbial source tracking (MST) methods are powerful tools to manage water quality and support public health risk assessment. However, their application is limited by the lack of specialized equipment and trained personnel in laboratories performing microbiological water quality assessment. Here, we describe a novel molecular method that combines helicase-dependent amplification (HDA) with a strip test for detecting ruminant faecal pollution sources. Unlike quantitative PCR (qPCR), the developed HDA-strip assay only requires a heating block to amplify the ruminant-associated *Bacteroidetes* 16S rRNA marker (BacR). Following HDA, the reaction mixture can be directly applied onto the test strip, which detects and displays the amplification products by marker-specific hybridization probes via an on-strip colorimetric reaction. The entire assay takes two hours and demands no extensive practical training. Furthermore, the BacR HDA-strip assay achieved comparable results in head-to-head performance tests with the qPCR reference, in which we investigated source-sensitivity and source-specificity, the analytical limit of detection, and the sample limit of detection. Although this approach only yields qualitative results, it can pave a way for future simple-to-use MST screening tools.

## Introduction

Faecal pollution of water is a serious problem that affects water systems in both developing and industrialized countries. Numerous pathogens transmitted through human and animal faeces can cause life-threatening diseases and economic losses. Understanding the origin of faecal pollution is essential not only for correctly assessing associated health risks, which vary and depend on faecal sources (human versus different animals), but also for devising effective management actions to prevent further pollution or remediate the causes. However, water resource managers and public health officials responsible for conducting monitoring and assessment of microbiological water quality have a limited number of tools available. Routine cultivation-based methods for standard faecal indicator bacteria enumeration (SFIB, e.g., *Escherichia coli* and intestinal enterococci)^1,2^ provide no information about pollution sources. Elevated levels of SFIB in environmental waters can originate from human wastewater, manure from livestock operations or diffuse sources such as input from wildlife and/or urban runoff. In addition, there are environmental habitats, such as terrestrial soils, aquatic sediments and aquatic vegetation, in which SFIB can persist and even naturally grow (non-faecal sources)^3–5^.

To address the limitations of the traditional SFIB approach, molecular microbial source tracking (MST) methods have been developed within the last decades that can link faecal pollution to its source^6–8^. These complementary and emerging tools can identify and discriminate different faecal pollution sources using the close association of certain faecal microorganisms with a specific host^7,9^. Bacteria of the order *Bacteroidales* are currently the most prominent faecal source identifiers in water^10^. These strictly anaerobic bacteria demonstrate certain host associations, are far more abundant in the faeces of mammals than SFIB, and the advent of molecular methods has allowed these non-culturable organisms to be used as alternative faecal indicators^11^. Polymerase chain reaction (PCR) and, especially, quantitative PCR (qPCR) have become the preferred methods for the detection and quantification of source-associated genetic markers in water samples^12,13^. To date, PCR-based assays have been developed to identify various potential faecal pollution sources, such as humans, ruminants, cattle, pigs, chickens, horses, elk, dogs, gulls and other birds (reviewed in^7,10^). However, the widespread use of these powerful tools is often limited. Many laboratories conducting water quality monitoring mainly with chemical and microbiological methods do not have access to the type of molecular biology equipment needed, such as costly high-precision qPCR thermocyclers. In addition to the technical demands, the establishment of molecular MST requires extensive technical training to perform the methods and interpret the results.

Isothermal DNA amplification methods, such as helicase-dependent amplification (HDA)^14^, can address these limitations of PCR technology by using alternative amplification strategies without the need for high-end PCR/qPCR instruments. Inspired by biological processes, HDA employs helicase enzymes to separate the strands of duplex DNA during the reaction, as opposed to heat denaturation over the course of thermal cycling in PCR^14^. This amplification strategy allows the method to work at a constant temperature on a standard heating block or in a water bath (~ 65°C) without sacrificing amplification efficiency. At the same time, the PCR-like reaction scheme of HDA (i.e., the use of two primers to flank the genetic marker) allows for the adaption of PCR assays to the HDA system^15,16^. This makes HDA an appealing method for settings with limited resources and infrastructure, especially when combined with paper-based analytical devices that enable rapid, simple and low-cost detection of amplification products^15,17^. Such nucleic acid lateral-flow strip tests rely on passive fluidics, can be easily manufactured and allow a user-friendly visual readout of test results that can be interpreted with the naked eye. Respective HDA-strip test systems have been proposed in PCR-dominated fields, such as clinical diagnostics (e.g.,^18–20^) and food safety testing (e.g.,^21–23^), with the intention of making molecular methods more accessible and relevant in point-of-need settings. Unfortunately, simplified molecular detection systems for environmental monitoring are rare^16,24–26^.

To provide a foundation for future, rapid, simple-to-use and low-cost molecular source tracking tools, we developed an HDA-strip assay to detect the ruminant-associated genetic marker (16S rRNA) in faecal members of the phylum *Bacteroides*. This marker – referred to as “BacR” – is traditionally determined by qPCR^27^ and has become one of the most widely used MST assays to detect ruminant faecal pollution sources in environmental waters. Thus, the specific aims were (i) to both design and develop a BacR HDA assay and a BacR strip test and (ii) to assess their combined performance in comparison to the qPCR reference method in experiments investigating source-sensitivity, source-specificity, the analytical limit of detection, and the sample limit of detection.

## Results and Discussion

**Figure 1:**
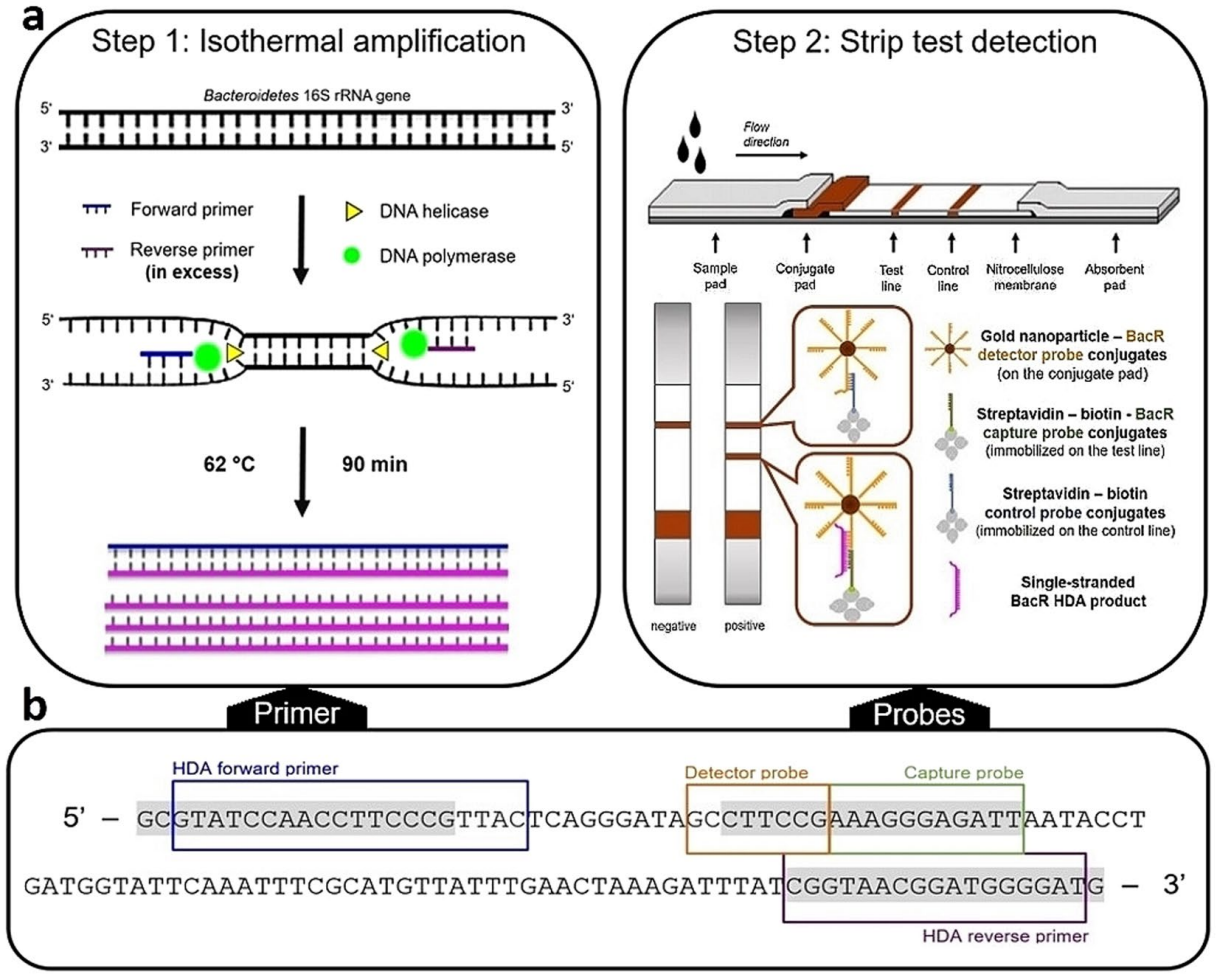
Schematic overview of the developed BacR HDA-strip assay (**a**) with incorporated HDA primer and strip test probe sequences (**b**). (**a**) (Step1) Asymmetric HDA amplification of the BacR marker on a heating block. (Step 2) Sequence-specific detection of HDA-derived single-stranded BacR marker copies via the nucleic acid lateral-flow strip test and visual readout of results. (**b**) Primer and probe binding sites of the HDA-strip and qPCR assay (GenBank accession number AF233400, bases 89 to 206). HDA primer and strip test probe regions (boxed) stretch over the qPCR primer and probe regions (marked in grey). The HDA amplicon is slightly shorter than the qPCR amplicon (115 bp versus 118 bp).

The principle of the developed BacR HDA-strip assay is schematically illustrated in Fig. 1. The method relies on an asymmetric HDA assay (i.e., one primer is used in excess concentration) that is entirely performed on a standard heating block and that produces single-stranded BacR copies once the limiting primer is used up (Fig. 1a, Step 1). These amplification products are then detected with the nucleic acid lateral-flow strip test by directly applying the final HDA mixture onto the sample application pad of the test strip (Fig. 1a, Step 2). Due to asymmetric HDA, no processing procedure (e.g., heat or chemical denaturation) is required between the amplification and the detection step to render the HDA amplicons single-stranded for subsequent hybridization reactions on the lateral-flow strip. Following sample application, the test strip only needs to be placed in running buffer (8x SSC, 0.1% Tween 20, 1% SDS), and after 15 min reaction time, the result can be visually read by observing the colouring of the test and control lines (Fig. 1a, Step 2).

### BacR HDA assay design and development

For the development of the BacR HDA assay, we initially tested whether we could use the original qPCR primers in HDA. However, HDA is more restrictive regarding primer design and amplicon selection than PCR^28^. For this reason, we needed to adapt both primer sequences to yield amplification products under isothermal conditions (Fig. 1b). A detailed comparison between the HDA and qPCR primer sets is given in Supplementary Fig. S1. Once the HDA primer set was designed, a series of experiments were performed in which we varied the concentration of both primers (50–500 µM), the reaction time (60–120 min) and the reaction temperature (60–65 °C) to ensure optimal asymmetric amplification conditions (data not shown). Final optimal reaction conditions are those described in the Materials and Methods section.

### BacR strip test design and development

Based on the work of Mao *et al*.^29^, the single-stranded BacR HDA products were captured and detected on the lateral-flow strip via sandwich DNA hybridization reactions. The strip test format therefore relied on two BacR marker-specific probes: a gold nanoparticle-labelled detector probe (deposited on the conjugate pad; AuNP-DP) and a biotinylated capture probe (immobilized on the membrane by binding to streptavidin; STV-Bio-CP), both illustrated in Fig. 1b. In positive reactions, the single-stranded BacR HDA products (amplified antisense strands) first hybridize with the AuNP-DP as the solution migrates along the strip. The AuNP-DP-amplicon hybrids are then drawn onto the membrane and are captured at the test line via a second sequence-specific hybridization reaction between the BacR HDA amplicon and the immobilized capture probes. Due to the accumulation of gold nanoparticles, these sandwich hybridization reactions are visible at the test line as a red-coloured line. Excess AuNP-DP migrate further and are captured at the control line via immobilized control probes. These control probes bind directly to the detector probe sequence, giving a second coloured line. The control line verifies a proper fluid flow and should appear in both positive and negative reactions, as its hybridization is independent of the presence of BacR HDA products. In contrast, the test line should only appear in positive reactions (i.e., with BacR markers amplified).

This nucleic acid lateral-flow assay format differs from most previously reported HDA-strip test systems, which usually capture hapten-labelled probe-amplicon hybrids on the test line with immobilized antibodies (e.g.,^18,30,31^). However, we aimed for a low-cost detection system with high analytical specificity to compensate for potential losses when adapting a qPCR-based MST method to an HDA system. As in the case of the BacR qPCR assay, a TaqMan minor-groove binder probe is used to detect the ruminant-associated *Bacteroidetes* marker^27^. In general, these probes are shorter than regular 5′ nuclease qPCR probes and offer increased analytical specificity^32,33^. To that end, we carefully designed detector and capture probes with relatively short marker-specific sequences (8 and 11 bases, with melting temperatures of ~36°C). Both probes bind at almost the same position as the BacR qPCR probe (Fig. 1b), but at room temperature and in lateral flow. In initial experiments, we tested the specificity of the developed strip test assay with a set of defined synthetic oligonucleotides (Supplementary Table S1). The oligonucleotides had a length of 115 bases to simulate single-stranded HDA products and only differed in a few singly mutated bases within the probe-binding regions. We observed that as soon as more than one base mismatched the probe sequences, the strip test result was negative (Supplementary Fig. S2).

### Source-sensitivity and source-specificity

We tested DNA extracts from faecal samples of known origin to determine whether the developed BacR HDA-strip assay can correctly detect ruminant faecal pollution sources. The sample set comprised a total of 20 faecal samples from ruminant livestock and wildlife, as well as 20 non-ruminant animal faecal samples including humans and representatives of omnivores, herbivores, carnivores, fish and birds to cover a range of faecal sources (Table 1). Each faecal DNA extract was analysed with both the HDA-strip assay and the qPCR assay, each in triplicate. As shown in Table 1, the results of the developed HDA-strip assay are almost identical to those of the qPCR assay. The HDA-strip assay correctly discriminated all ruminant from non-ruminant faecal samples (only one false-positive result was produced in a single replicate analysis of the wild boar sample). Exemplary results of the HDA-strip assay are shown in Supplementary Fig. S3. This performance suggests that the adaptation of the qPCR primers and probe to the HDA-strip test platform had no adverse effects on the diagnostic utility of the assay.

**Table 1:** Source-sensitivity and source-specificity results of the BacR HDA-strip assay and the BacR qPCR assay.

Ruminants				Non-Ruminants			
Source	Scientific name	HDA- strip	qPCR^a^	Source	Scientific name	HDA-strip	qPCR
Cattle 1	*Bos Taurus*	3/3	3/3	Human 1	*Homo sapiens*	0/3	0/3
Cattle 2	*Bos Taurus*	3/3	3/3	Human 2	*Homo sapiens*	0/3	0/3
Cattle 3	*Bos Taurus*	3/3	3/3	Human 3	*Homo sapiens*	0/3	0/3
Cattle 4	*Bos Taurus*	3/3	3/3	Human 4	*Homo sapiens*	0/3	0/3
Red sheep 1	*Ovis aries*	3/3	3/3	Pig	*Sus scrofa domesticus*	0/3	0/3
Red sheep 2	*Ovis aries*	3/3	3/3	Wild Boar	*Sus scrofa*	1/3	0/3
Red sheep 3	*Ovis aries*	3/3	3/3	Horse	*Equus caballus*	0/3	0/3
Red sheep 4	*Ovis aries*	3/3	3/3	Horse	*Equus caballus*	0/3	0/3
Goat 1	*Capra hircus*	3/3	3/3	Chicken	*Gallus gallus*	0/3	0/3
Goat 2	*Capra hircus*	3/3	3/3	Red Fox	*Vupes vulpes*	0/3	0/3
Red deer 1	*Cervus elaphus*	3/3	3/3	Dog	*Canis familiaris*	0/3	0/3
Red deer 2	*Cervus elaphus*	3/3	3/3	Cat	*Felis catus*	0/3	0/3
Red deer 3	*Cervus elaphus*	3/3	3/3	Beaver	*Castor fibre*	0/3	0/3
Red deer 4	*Cervus elaphus*	3/3	3/3	Common carp	*Cyprinus carpio*	0/3	0/3
Roe deer 1	*Capreolus capreolus*	3/3	3/3	Zander	*Sander luciperca*	0/3	0/3
Roe deer 2	*Capreolus capreolus*	3/3	3/3	Common bream	*Abramis brama*	0/3	0/3
Chamois 1	*Rupicapra rupicapra*	3/3	3/3	Brook trout	*Salvelinus fontinalis*	0/3	0/3
Chamois 2	*Rupicapra rupicapra*	3/3	3/3	Greylag goose	*Anser anser*	0/3	0/3
Ibex 1	*Capra ibex*	3/3	3/3	Grey heron	*Ardea cinerea*	0/3	0/3
Ibex 2	*Capra ibex*	3/3	3/3	Duck	*Anas platyrhynchos*	0/3	0/3
Source-sensitivity^b^		100%	100%	Source-specificity^c^		98%	100%

Assays were evaluated on 20 single ruminant (target) and 20 single non-ruminant (non-target) faecal samples, each tested at a concentration of 1 ng extracted DNA per reaction. The results are given as the number of positive reactions from triplicate analyses and as calculated sensitivity and specificity percentages.

^a^Marker copy numbers ranged from 10^4^ to 10^5^ copies per reaction based on qPCR analysis with plasmid standards.

^b^Source sensitivity (%): (true positives)/(true positives + false negatives) × 100.

^c^Source specificity (%): (true negatives)/(true negatives + false positives) × 100.

### Analytical limit of detection (LOD_95%_)

**Figure 2:**
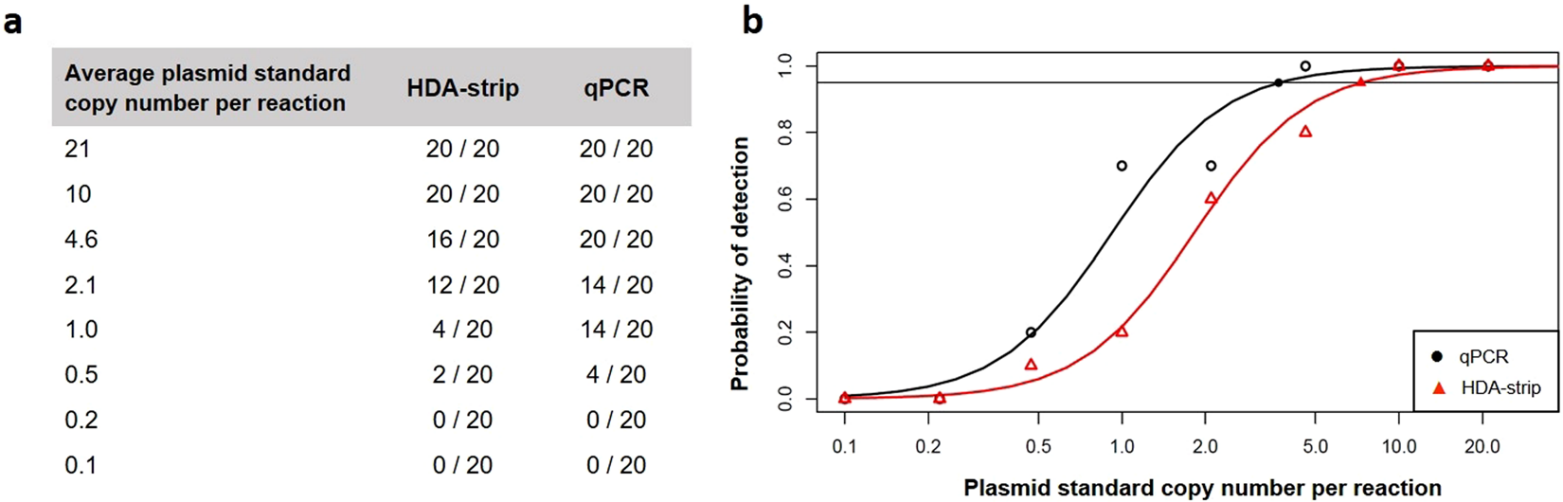
Limits of detection (LOD_95%_). (**a**) Raw data from the analyses of a dilution series of the BacR plasmid standard that served as the input for statistical calculations. (**b**) Logistic regression model used to determine the LOD_95%_, which is indicated by filled symbols on the horizontal line. LOD_95%_ (with 95% confidence interval): HDA = 5.2 ≤ 7.3 ≤ 10.3 copies; qPCR = 2.6 ≤ 3.7 ≤ 5.2 copies.

We used a defined plasmid standard carrying a single copy of the BacR marker to determine the analytical limit of detection (LOD_95%_). To this end, we serially diluted the plasmid standard to concentrations ranging from 21 to 0.1 copies per reaction and analysed the dilution series with both the HDA-strip assay and the qPCR assay in 20 replicates each. The results were statistically analysed with R software using the logistic regression model (Fig. 2). We determined an LOD_95%_ of 7.3 copies per reaction for the HDA-strip assay (95% confidence range: 5.2–10.3 copies) and 3.7 copies per reaction for the qPCR assay (95% confidence range: 2.6–5.2 copies). Considering the differences in instrument complexity, the HDA-strip assay has shown excellent performance comparable to that of the qPCR assay. Moreover, we observed no decline in the intensity of the characteristic red test line of the HDA-test strip with decreasing template plasmid copy numbers per reaction (Supplementary Fig. S4). These results suggest a highly efficient adaptation and optimization of the HDA-strip assay conditions.

### Sample limit of detection (SLOD)

To determine the lowest possible concentration of faecal material detectable in water, we generated faecal suspensions from three important ruminant species (cattle, sheep and fallow deer) and serially diluted these suspensions in river water as the environmental matrix of interest. Each of the faecal sample dilutions (10^−2^, 10^−3^, 10^−4^, 10^−5^ and 10^−6^) was taken through the entire sample processing procedure, i.e., sample concentration on membrane filters and DNA extraction with phenol/chloroform. The extracted sample DNAs from two independent filtrations were then analysed with both the HDA-strip and qPCR assay in ten replicates each. The obtained results served as input for statistical analysis. The logistic regression model (R software) was used to determine the lowest detectable concentration of ruminant faeces at 95% confidence (SLOD_95%_) for each of the ruminant species and detection methods.

The results are summarized in Table 2. The performance of the developed HDA-strip assay was highly similar to that of qPCR. The average SLOD_95%_ of ruminant faecal material was 6.2 * 10^−8^ g faeces per analysed filter for the HDA-strip assay and 6.0 * 10^−8^ g faeces per analysed filter for the qPCR assay. Exemplary results of the HDA-strip assay are shown in Supplementary Fig. S5.

**Table 2:** Sample limit of detection experiments with the HDA-strip and qPCR method.

Source	Faecal dilution	HDA-strip		qPCR		Detected concentration (10^-8^ g faeces per filter)			
		Filter 1	Filter 2	Filter 1	Filter 2	HDA-strip		qPCR	
						SLOD_95%_	95% CI	SLOD_95%_	95% CI
Cattle	10^−2^	10/10	10/10	10/10	10/10	4.9	2.4–10.4	2.8	1.3–5.9
	10^−3^	10/10	9/10	10/10	10/10				
	10^−4^	4/10	8/10	5/10	10/10				
	10^−5^	0/10	2/10	1/10	2/10				
	10^−6^	0/10	0/10	0/10	0/10				
Sheep	10^−2^	10/10	10/10	10/10	10/10	2.5	1.2–5.2	2.5	1.2–5.2
	10^−3^	10/10	10/10	10/10	10/10				
	10^−4^	4/10	10/10	6/10	10/10				
	10^−5^	1/10	4/10	1/10	2/10				
	10^−6^	0/10	0/10	0/10	0/10				
Fallow Deer	10^−2^	10/10	10/10	10/10	10/10	11.3	4.9–25.9	12.6	5.5–29.1
	10^−3^	9/10	9/10	9/10	10/10				
	10^−4^	0/10	8/10	1/10	7/10				
	10^−5^	1/10	2/10	0/10	1/10				
	10^−6^	0/10	0/10	0/10	0/10				

The results are given as the number of positive replicates from the analyses of 10 replicates per faecal dilution step. The total number of replicates from both filtrations (i.e., n = 20 replicates per faecal dilution) served as input for statistical analysis. Detection limits were calculated as the minimum amount of faeces detectable in a filtration volume at a 95% detection probability (SLOD_95%_) with a 95% confidence interval (CI).

### Perspectives and outlook for the developed BacR HDA-strip assay

Given its simplicity and excellent performance, the developed HDA-strip assay provides a promising alternative molecular diagnostic tool for identifying ruminant faecal sources in water. Unlike its qPCR counterpart, it relies on an instrument that is readily available in microbiology laboratories (standard heating block). Likewise, the results are easy to read and interpret by non-experts. However, both assays (HDA-strip and qPCR) still require water concentration based on membrane filtration and DNA extraction from filters resulting in a similar overall sample testing time of approximately three hours. Unfortunately, these traditional upstream processes constitute barriers for the application of the HDA-strip assay in resource-limited settings. Thus, further research activities need to tackle the development of rapid and simplified sample preparation procedures. For instance, filter-back flush sampling methodology using syringe filters^25^ and ionic liquid-based DNA extraction^34^ could be promising strategies to build up an entire workflow when combined with the developed HDA-strip assay. In addition, these attempts could form the basis for fully integrated and portable devices for point-of-need applications in remote areas. Such a system was recently demonstrated by Tang *et al*.^23^, who developed an inexpensive and battery-operated device with sample-in-answer-out functionality that consisted of three modules, namely, nucleic acid extraction, an HDA module and a lateral-flow strip detection module^23^. For any such attempts, the developed HDA-strip assay provides a fundament which is not restricted to the detection of the ruminant-associated *Bacteroidetes* marker chosen to demonstrate this novel approach.

Several host-associated PCR and qPCR assays are currently available for faecal source identification in water. For this proof-of-concept study, we selected the BacR marker and its respective qPCR assay as a performance benchmark since the marker was initially developed in our laboratory and has been evaluated and applied in several field studies conducted by our group as well as by other colleagues in the MST field^35–40^. Furthermore, the results from a geographically expansive comparison study that spanned 16 countries and six continents suggested that the BacR marker was robust worldwide, while human markers seem to be less prevalent and stable in some regions of the world and could be improved for global applications^41^. These aspects made the BacR qPCR assay an ideal candidate method and starting point for simplification efforts.

A general limitation of the developed HDA-strip assay approach is that it yields qualitative (presence/absence) information. Microbial source tracking based on qPCR provides quantitative levels of source-associated markers, which is highly useful for pollution source identification and risk assessment. Unfortunately, the threshold for implementation of MST methods is currently very high and therefore quite limited. Isothermal amplification assays, such as the one developed in this study, have the potential to allow much more widespread application. In spite the fact that the HDA-strip approach provides only qualitative information for MST markers, its simplicity and high performance (LOD, source-specificity) makes it a viable option for source identification in settings that lack the specialized equipment and trained personnel required to perform qPCR-based methods. In this way, watershed managers would be able to cost-effectively test for the presence or absence of faecal pollution sources (e.g., to back up the results of sanitary surveys or to identify the primary faecal pollution sources, especially when multiple assays targeting different genetic markers are performed on the same DNA extract). Such an approach would allow easier movement from the sampling and monitoring of environmental waters to water quality management. In addition, it would provide an opportunity for developing countries to use and profit from state-of-the-art molecular-based MST technology, which is currently mostly inaccessible in those regions. Considering that severe pathogen pollution already affects around one-third of all river stretches in Africa, Asia and Latin America^42^, there is an urgent need for tools that enable both detection and characterization of faecal pollution to better manage water quality and public health.

## Conclusion

In this study, we developed the first HDA-strip assay for identifying faecal pollution sources in water by targeting one of the most prominent microbial source tracking markers, BacR. Due to careful assay design and development, we achieved almost identical results with the HDA-strip assay in head-to-head performance tests with its qPCR reference method. Thus, we conclude that this amplification and detection platform provides a complementary tool to the BacR qPCR assay (regarding equivalence in source-specificity and sensitivity, as well as detection limits) but without the current limitations of cost and technical complexity. We anticipate that such novel tools can help move microbial source tracking activity towards wider applications, especially when further genetic markers and assays are adapted to isothermal amplification reaction schemes.

## Materials and Methods

### Faecal sample processing for performance tests

For source-sensitivity and source-specificity experiments, single ruminant and non-ruminant faecal samples were collected in eastern Austria in sterile 50 mL polypropylene sampling tubes (stored at −20 °C until further processing). DNA was extracted using the PowerSoil^TM^ DNA isolation kit (MO BIO Laboratories, Carlsbad, CA) according to the manufacturer’s instructions. DNA extracts were stored at −80 °C. DNA concentrations were measured using the QuantiFluor dsDNA Dye System (Promega, Mannheim, Germany). All analysed DNA extracts contained amplifiable DNA, as determined with the AllBac qPCR assay (general *Bacteroidetes* 16S rRNA gene marker)^43^. Sample dilutions were judged as free of inhibition based on matching concentrations between two dilutions, as described previously^44^.

For sample limit of detection (SLOD) experiments, pooled faecal samples were created that consisted of 10 single samples of the same ruminant source (cattle, sheep and fallow deer, respectively; freshly collected and stored at 4 °C). Faecal suspensions were prepared in surface water sampled from the Danube river in a sterile 500-mL glass bottle (transported to the laboratory in a cooling box and kept at 4 °C until processing). In brief, 500 ± 10 mg of pooled and homogenized faeces were immediately suspended in 45 mL surface water in 50-mL centrifugation tubes (Sterilin, Aberbargoed, UK) by vortexing the tubes for 10 s each. The tubes were then incubated at 4 °C for one hour. After gentle vortexing and sedimentation of plant residues (10 s each step), the faecal suspensions were diluted 100-fold (i.e., 10^−2^), followed by serial 10-fold dilutions (i.e., 10^−3^, 10^−4^, 10^−5^, and 10^−6^). One mL of each dilution was immediately filtered through polycarbonate filters (0.2-µM pore size, 45-mm diameter; Millipore, Bedford, MA). Each filter was aseptically folded several times and placed in a 1.5-mL Eppendorf tube for DNA extraction (stored at −80 °C until extraction). Two independent filtrations were performed for each sample dilution. DNA extraction from filters was performed using bead beating and phenol/chloroform, as described by Griffith *et al*.^45^, with DNA precipitation using isopropanol instead of polyethylene glycol. Recovered DNA was dissolved in 100 µL of 10 mM Tris-HCl, pH 8, and stored at −80 °C. Filtration blanks (surface water without faecal material added) and extraction blanks (filters without faecal suspension added) were processed in parallel to test for contamination, and all tested negative. Prior to analysis, all DNA extracts were 4-fold diluted in a 5-µg mL^−1^ poly-dIdC solution as a non-specific DNA background (Roche Diagnostics, Mannheim, Germany).

### Primer and probe sequences

All oligonucleotide sequences were synthesized by Sigma-Aldrich (Steinheim, Germany). Primer and probe sequences for qPCR, HDA and the nucleic acid lateral flow strip test are given in Table 3. qPCR primer and probe sequences were previously published by our group^27^. HDA primers were designed using the Primer3 4.1 programme (http://primer3.ut.ee), whereas nucleic acid lateral-flow strip test probes were designed and assessed with OligoAnalyzer 3.1 (https://eu.idtdna.com/calc/analyzer).

**Table 3:** Oligonucleotides used in this study.

Name	Function	Sequence (5′–3′)^a^	Reference	Binding positions ^b^
H-BacR_f	HDA forward primer	GTATCCAACCTTCCCGTTAC	This study	91–110
H-BacR_r	HDA reverse primer	ATCCCCATCCGTTACCG	This study	189–205
H-BacR_CP	Strip test capture probe	AAAGGGAGATT(A)_20_-BtnTg	This study	120–127
H-BacR_DP	Strip test detector probe	ThiC6-(A)_20_GCCTTCCG	This study	128–138
H-BacR_CL	Strip test control probe	Btn-(A)_20_GAAGGCTTTTT	This study	—
BacR_f	qPCR forward primer	GCGTATCCAACCTTCCCG	^27^	89–106
BacR_r	qPCR reverse primer	CATCCCCATCCGTTACCG	^27^	189–206
BacR_p	qPCR probe	FAM-CTTCCGAAAGGGAGATT-NFQ-MGB	^27^	122–138

^a^Abbreviations: FAM, 6-carboxyfluorescein; NFQ, non-fluorescent quencher; MGB, minor groove binder; Btn/BtnTg, Biotin; ThiC6, Thiol group. (A)_20_ is a sequence of 20 oligo(dA) used as a spacer. ^b^Binding positions refer to GenBank accession number AF233400.

### Nucleic acid lateral-flow test strip fabrication

The nucleic acid lateral-flow test strips consisted of four components: the sample application pad (glass fibre, GFCP103000), the conjugate pad (glass fibre, GFCP103000), the membrane card (laminated card with adhered nitrocellulose membrane, HF180MC100), and the absorbent pad (cellulose, CFSP173000), which were all obtained from Millipore (Billerica, MA, USA). Details on the preparation of AuNP (gold nanoparticles), AuNP-DP (functionalized gold nanoparticle with DNA detector probes) and streptavidin-biotin-probe conjugates (STV-Bio-capture probe and STV-Bio-control probe) can be found in the Supplementary Information.

Prior to the assembly of strip components, the conjugate pad (8 × 300 mm) was prepared by dispensing AuNP-DP conjugates at a rate of 25 µL cm^−1^ onto the glass fibre pad using the Airjet Sprayer from BioDot (Irvine, CA, USA). Furthermore, specific probes for the test and control lines were sprayed onto the nitrocellulose membrane (25 × 300 mm) in form of streptavidin-biotin-probe conjugates. The test-line capture probe conjugates and the control-line control probe conjugates were deposited at a flow rate of 0.75 µL cm^−1^ using the Biojet Sprayer from BioDot. The distance between the test and control lines was approximately 5 mm. After spraying, conjugate pads and membranes were dried for two hours at 37 °C. Finally, the absorbent pad, conjugate pad and sample pad were sequentially mounted on the adhesive membrane card with overlaps of 2 mm to ensure a continuous flow path of the sample. Test strips of 6-mm width were cut with the BioDot guillotine cutter and were stored in a desiccator at room temperature until use.

To ensure optimal nucleic acid lateral-flow assay conditions, a series of experiments was performed to determine the most efficient dispensing volume of AuNP-DP for immobilization on the conjugate pad (10–40 µL cm^−1^), the dispensing volume of STV-Bio-probe conjugates on the membrane (0.5–1.25 µL cm^−1^) and their dilution factor prior to dispensing (1:2–1:10). In addition, the running buffer composition (2x–10x SSC with Tween20 and SDS in different concentrations ranging from 0.1–10%) and assay time (5–20 min) were tested.

### BacR HDA-strip test assay protocol

BacR HDA reactions were performed on a heating block (PocketBloc, Biozym, Germany), using the IsoAmp II Universal tHDA kit (New England Biolabs, Ipswich, MA) in a final reaction volume of 20 µL. Following a two-step protocol, 10 µL of Mix A containing 1x annealing buffer II, 100 nM H-BacR_f, 300 nM H-BacR_r, and 2.5 µL DNA template was overlaid with mineral oil and heated at 95 °C for 3 min for initial DNA denaturation. After subsequent equilibration at 62 °C for 3 min, 10 µL of Mix B containing 1x annealing buffer II, 8 mM MgSO_4_, 80 mM NaCl, 3.5 µL of IsoAmp dNTP solution, and 3.5 µL of IsoAmp enzyme mix was added. Reaction mixtures were then incubated for 90 min at 62 °C. HDA products were visualized by transferring 10 µL of the HDA reaction onto the sample application pad of the developed nucleic acid lateral-flow test strip. After placing the test strip in 250 µL running buffer (8x SSC, 0.1% Tween 20, 1% SDS; reagents purchased from Sigma Aldrich) and incubation for 15 min, the results were visually evaluated. Based on the presence or absence of a coloured test line on the test strip, HDA-strip assay results were scored as positive or negative, respectively. Unless otherwise noted, all amplification reactions were performed in triplicate, including no-template controls (NTC) in each run to check for contamination.

### BacR qPCR assay protocol

BacR qPCR reactions were performed in a total reaction volume of 15 µL containing 1x Kapa Probe Fast (PeqLab, Erlangen, Germany), 100 nM BacR_f, 500 nM BacR_r, 100 nM BacR_p, and 2.5 µL of DNA template. The amplification reactions were run on a 7500 Fast Real-Time PCR system (Applied Biosystems, New York, NY) according to the following thermal cycling conditions: an initial step of 5 min at 95 °C, followed by 45 cycles of 15 s at 95 °C and 15 s at 60 °C. Unless otherwise noted, all amplification reactions were performed in triplicate, including no-template controls (NTC) in each run to check for contamination. The results of qPCR were scored negative when the c_q_ value was undetermined or <1 copy was detected. Quantification was based on dilutions of a plasmid standard carrying a single copy of the BacR marker sequence. Plasmid standard dilutions were prepared in an unspecific background of 500 µg L^−1^ poly(dI-dC) (Roche Diagnostics, Mannheim, Germany) to avoid adsorption of plasmid DNA onto the reaction tubes at low concentrations. A total of seven tenfold serial dilutions of plasmid standard (10^0^–10^6^ marker copies per reaction) was included in each qPCR run. In addition, DNA sample dilutions were measured in each qPCR run and were judged free of inhibition based on matching concentrations between two dilutions, as described previously^44^.

### Data analyses

The source-sensitivity and -specificity of each method (reported as percentage) were calculated based on the following mathematical equations^46^: sensitivity (%) = TP/(TP + FN) × 100, where ‘TP’ is the number of positive samples and ‘FN’ is the number of false negatives when ruminant samples were used, and specificity (%) = TN/(TN + FP) × 100, where ‘TN’ is the number of negative samples and ‘FP’ is the number of false positives when non-ruminant faecal samples were used. A 95% limit of detection (LOD_95%_) was defined as the concentration at which a detection probability of 95% is expected. Detection probabilities were modelled as a function of concentration using a logistic regression model. R software (R Development Core Team, 2008) was employed for this computation.

## Electronic supplementary material

Supplementary Information

## Data Availability

The datasets generated during and/or analysed during the current study are available from the corresponding author on reasonable request.
