# Supplementary material for: Detection of a microbial source tracking marker by isothermal helicase-dependent amplification and a nucleic acid lateral-flow strip test
Source: Sci Rep. 2019 Jan 23;9:393. doi: 10.1038/s41598-018-36749-7 (PMC6344534; doi:10.1038/s41598-018-36749-7)
Supplement: Supplementary file 1 — Supplementary Information [file 41598_2018_36749_MOESM1_ESM.pdf]

Supplementary Information

Detection of a microbial source tracking marker by isothermal helicase-dependent amplification and a nucleic acid lateral flow strip test

Claudia Kolm[1,2], Roland Martzy[1,2], Manuela Führer[3], Robert L. Mach[4], Rudolf Krska[3,5], Sabine Baumgartner[3], Andreas H. Farnleitner[2,6,7], Georg H. Reischer[1,7]*

[1] TU Wien, Institute of Chemical, Environmental & Bioscience Engineering, Molecular Diagnostics Group, Department IFA-Tulln, Tulln, Austria; [2] ICC Interuniversity Cooperation Centre Water & Health, Vienna, Austria (www.waterandhealth.at); [3] University of Natural Resources and Life Sciences, Vienna (BOKU), Department IFA-Tulln, Center for Analytical Chemistry, Tulln, Austria; [4] TU Wien, Institute of Chemical, Environmental & Bioscience Engineering, Research Division Biochemical Technology, Research Group Synthetic Biology and Molecular Biotechnology, Vienna, Austria; [5] Queen's University Belfast, School of Biological Sciences, Institute for Global Food Security, Northern Ireland, United Kingdom; [6] Karl Landsteiner University of Health Sciences, Research Unit Water Quality and Health, Krems, Austria; [7] TU Wien, Institute of Chemical, Environmental & Bioscience Engineering, Research Division Biochemical Technology, Research Group of Environmental Microbiology and Molecular Diagnostics, Vienna, Austria;

* To whom correspondence should be addressed. Tel: [+43158801166556]; Email: [georg.reischer@tuwien.ac.at].

CONTENT:

1. HDA primer design
2. Nucleic acid lateral-flow strip test experiments with synthetic oligonucleotides
3. Performance tests
4. Preparation of gold nanoparticles (AuNP)
5. Preparation of gold nanoparticles-detector probe conjugates (AuNP-DP)
6. Preparation of streptavidin-biotin-DNA conjugates (H_BacR-CP and H_BacR-CL)

1. HDA primer design

A

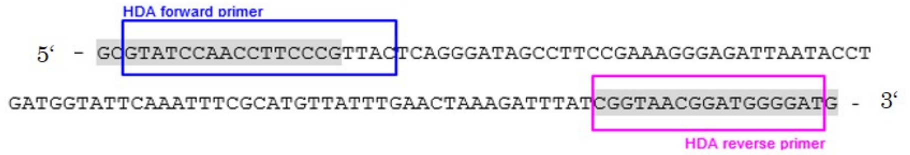

B

		Primer			Amplicon	
		bp	GC	Tm	bp	Tm
Recommended parameter ranges for HDA:		24-33	35-60%	60-74°	70-120	68-77°
BacR qPCR primer	Forward<br>Reverse	18<br>18	61%<br>61%	63.0°<br>63.5°	118	77.3°
BacR HDA primer	Forward<br>Reverse	17<br>20	59%<br>50%	62.0°<br>61.2°	115	76.5°

C

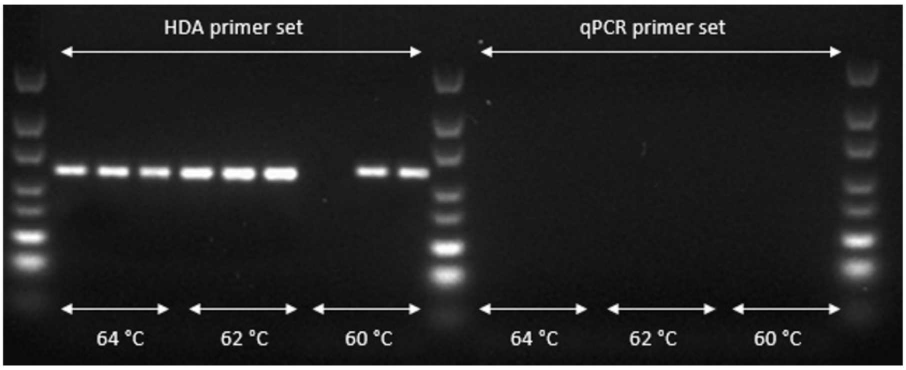

Supplementary Figure S1. (A) Sites of the BacR HDA and BacR qPCR primer binding (GenBank accession number AF233400): qPCR primer binding sites are marked in grey, HDA primer binding sites are boxed. While qPCR targets a 118-bp long marker region from position 89 to 206, HDA targets a 115-bp long marker region from position 91 to 205. (B) Primer and amplicon parameters of the HDA and qPCR assay, with specific recommendations given for HDA. Although the criteria do not match perfectly, HDA primers yielded amplification products under isothermal conditions. (C) Results from initial HDA experiments in which the designed HDA and the original qPCR primers were both tested on a DNA extract from cattle faeces (0.1 ng DNA per reaction). Reactions were performed in triplicate at three different temperatures (60 °, 62 °, 64 °C) using an equal primer ratio (final concentration of 50 nM each; symmetric format). HDA products were analysed on a 2.8% agarose gel stained with SYBR Gold.

2. Nucleic acid lateral-flow strip test experiments with synthetic oligonucleotides

Supplementary Table S1. The specificity of the developed strip test was challenged in experiments with defined synthetic oligonucleotides (chemically synthesized by Sigma-Aldrich). In total, we designed and experimentally tested nine different single-stranded oligos (SO1-9), all with a length of 115 bases, to simulate asymmetrically amplified HDA products. These oligos differed only by a few bases (indicated in red) within the binding region of the strip test probes (marked in grey). Apart from the single-base mutations, however, the rest of the oligo sequence was identical to that of the BacR 16S rRNA marker (GenBank accession number AF233400, position 91 to 205 including primer binding sites). Oligo SO1 was used as a positive control (sequence is identical to position 91 to 205, acc.no. AF233400).

Synthetic oligo	5' – 3' (detector probe and capture probe binding sites)
SO1	GTATCCAACCTTCCCGTTACTCAGGGATAGCCTTCCGAAAGGGAGATTAATACCTGATGGTATTCAAATT<br>TCGCATGTTATTTGAACTAAAGATTTATCGGTAACGGATGGGGAT
SO2	GTATCCAACCTTCCCGTTACTCAGGGATAGCCTCCCGAAAGGGAGATTAATACCTGATGGTATTCAAATT<br>TCGCATGTTATTTGAACTAAAGATTTATCGGTAACGGATGGGGAT
SO3	GTATCCAACCTTCCCGTTACTCAGGGATAGCCTGCCGAAAGGCAGATTAATACCTGATGGTATTCAAATT<br>TCGCATGTTATTTGAACTAAAGATTTATCGGTAACGGATGGGGAT
SO4	GTATCCAACCTTCCCGTTACTCAGGGATAGCCCGTCGAAAGGCGGATTAATACCTGATGGTATTCAAATT<br>TCGCATGTTATTTGAACTAAAGATTTATCGGTAACGGATGGGGAT
SO5	GTATCCAACCTTCCCGTTACTCAGGGATAGCCTTTCGAAAGAAAGATTAATACCTGATGGTATTCAAATT<br>TCGCATGTTATTTGAACTAAAGATTTATCGGTAACGGATGGGGAT
SO6	GTATCCAACCTTCCCGTTACTCAGGGATAGCCTTGCGAAAGTAAGACTAATACCTGATGGTATTCAAATT<br>TCGCATGTTATTTGAACTAAAGATTTATCGGTAACGGATGGGGAT
SO7	GTATCCAACCTTCCCGTTACTCAGGGATAGCCCGGTGAAAACCGGACTAATACCTGATGGTATTCAAATT<br>TCGCATGTTATTTGAACTAAAGATTTATCGGTAACGGATGGGGAT
SO8	GTATCCAACCTTCCCGTTACTCAGGGATAGCCTCGGGAAACTGAGAGTAATACCTGATGGTATTCAAATT<br>TCGCATGTTATTTGAACTAAAGATTTATCGGTAACGGATGGGGAT
SO9	GTATCCAACCTTCCCGTTACTCAGGGATAGCCTCCTGAAAGGGAGTTTAATACCTGATGGTATTCAAATT<br>TCGCATGTTATTTGAACTAAAGATTTATCGGTAACGGATGGGGAT

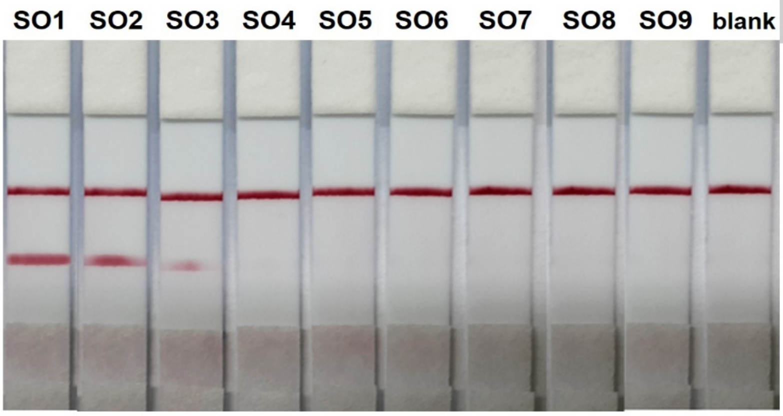

Oligo (SO)	Detector probe binding site	Capture probe binding site	Test strip result
1	GCCTTCCG	AAAGGGAGATT	3 / 3
2	GCCTCCCG	AAAGGGAGATT	3 / 3
3	GCCTGCCG	AAAGGCAGATT	3 / 3
4	GCCCGTCG	AAAGGCGGATT	0 / 3
5	GCCTTTCG	AAAGAAAGATT	0 / 3
6	GCCTTGCG	AAAGTAAGACT	0 / 3
7	GCCCGGTG	AAAACCGGACT	0 / 3
8	GCCTCGGG	AAACTGAGAGT	0 / 3
9	GCCTCCTG	AAAGGGAGTTT	0 / 3
-	Blank: 50 mM PB buffer, pH 8		0 / 3

Supplementary Figure S2. Strip test results from specificity experiments with synthetic oligonucleotides (SO1-SO9). For experimental testing, 10 µL aliquots of the oligonucleotides (containing ~ 10^{13} molecules) were directly applied onto the sample pad of the test strip (no amplification step prior). The test strips were then placed in 250 µL running buffer (8x SSC, 0.1% Tween 20 and 1% SDS; reagents purchased from Sigma-Aldrich), and the results were read after 15 min assay time. Base mutations in the respective binding sites of the strip test probes are indicated in red. The results are given as the number of positive test strips per triplicate analysis. In addition to the positive control (SO1), colouring of the test line was only observed in cases of single non-complementary bases (SO2 and SO3). As soon as more than one base mismatched, the strip test was negative (SO4 - SO9).

3. Performance tests

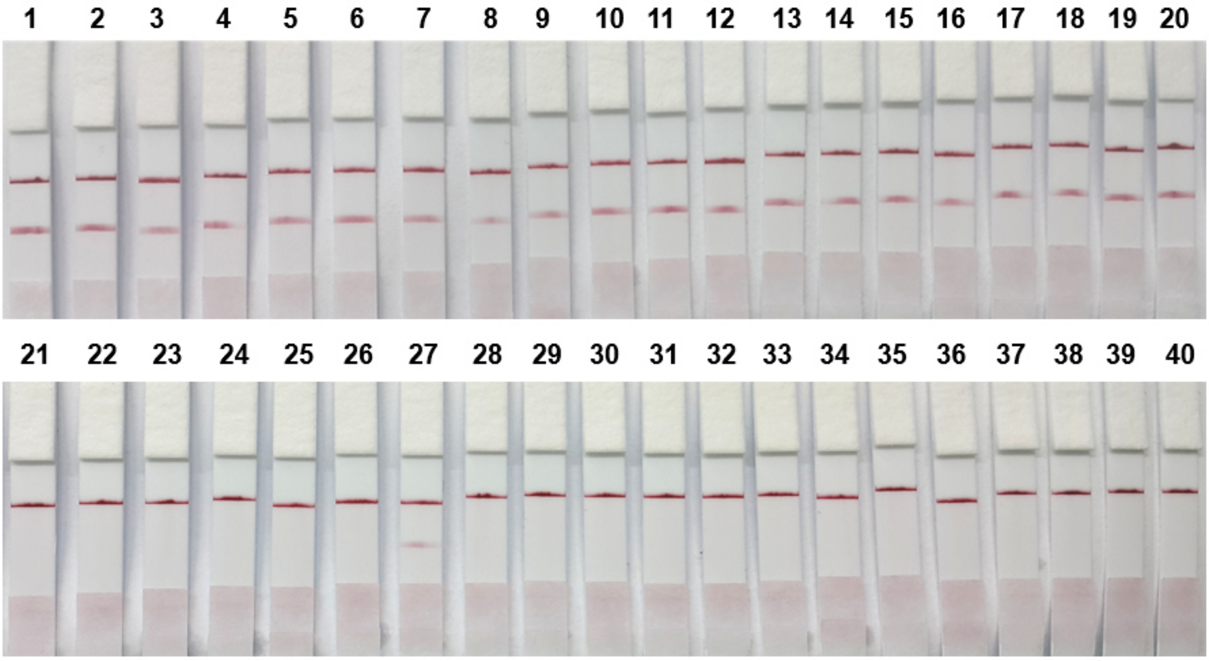

Supplementary Figure S3. Exemplary results of the BacR HDA-strip assay from source-sensitivity and source-specificity experiments testing DNA extracts from 20 ruminant faecal samples (1-20) and 20 non-ruminant faecal samples (21-40), respectively. *1-4* cattle, *5-8* red sheep, *9-10* domestic goat, *11-14* red deer, *15-16* European roe, *17-18* alpine chamois, *19-20* alpine ibex, *21-24* human, *25-26* horse, *27* wild boar, *28* domestic pig, *29* chicken, *30* red fox, *31* dog, *32* cat, *33* Eurasian beaver, *34* common carp, *35* common bream, *36* brook trout, *37* greylag goose, *38* grey heron, *39* mallard duck, *40* water rail. Due to the presence of a coloured test line, samples “1 to 20” and “27” were scored positive.

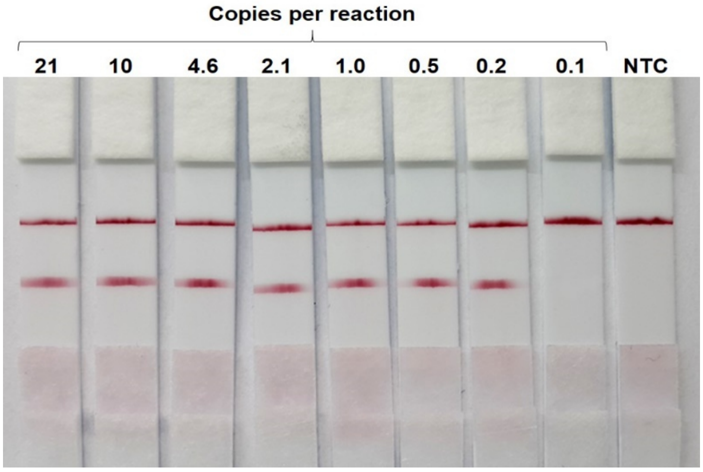

Supplementary Figure S4. Exemplary results of the BacR HDA-strip assay from analytical limit of detection ($LOD_{95\%}$) experiments analysing plasmid standard dilutions ranging from 21 to 0.1 copies per reaction. *NTC* no-template control

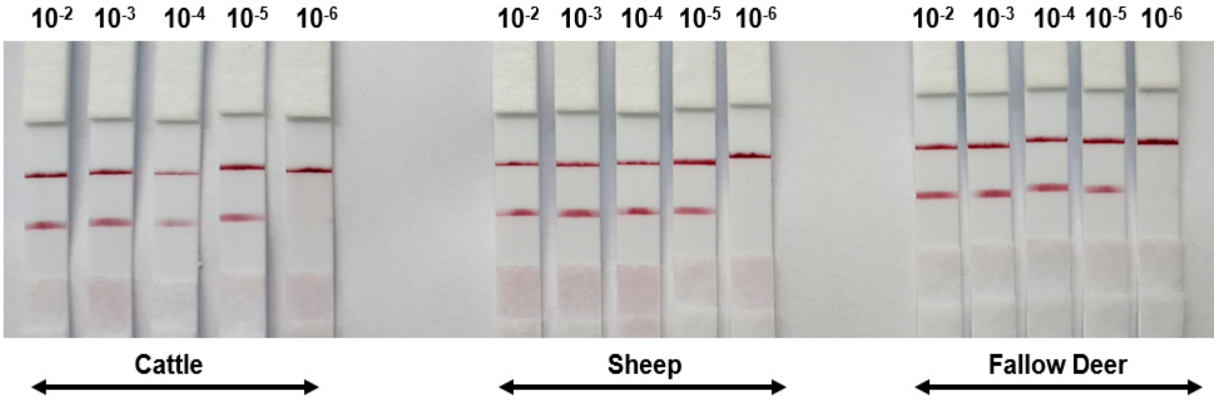

Supplementary Figure S5. Exemplary results of the BacR HDA-strip assay from sample limit of detection (SLOD) experiments analysing faecal ruminant suspensions from cattle, sheep and fallow deer. Pooled faeces samples (n = 10) were suspended and serially diluted from 10^{-2}-fold to 10^{-6}-fold with surface water.

4. Preparation of gold nanoparticles (AuNP)

Gold nanoparticles (AuNP) with an average diameter of 13 nm were prepared by controlled reduction of tetracholorauric (III) acid trihydrate ($HAuCl_4$ * $3H_2O$, Sigma Aldrich, St. Luis, MO, USA) with citric acid trisodium salt, following a modified protocol of the Turkevich-Frens method [1,2]. All glassware used was cleaned with aqua regia (three parts HCl, one part HNO_3), rinsed with ultrapure water (Milli-Q, 18.2 MΩ) and employed only for preparing colloidal gold solutions. In brief, 100 mL of a 0.039% $HAuCl_4$ solution (w/w) was brought to boil in a 250-mL round-bottom flask under reflux condition. The reduction process from Au^{3+} to Au^0 was initiated by adding 50 mL of a 38.8 mM sodium citrate solution under constant stirring. After colour change from yellow to deep-blue and, finally, dark-red, the solution was allowed to boil for another 10 min to complete the reduction. The heating source was then removed, and the solution was cooled to room temperature with stirring. The AuNP solution was stored at 4° C until preparation of AuNP-DNA detector probe conjugates.

5. Preparation of gold nanoparticle-detector probe conjugates (AuNP-DP)

AuNP were functionalized with thiolated detector probes (H-BacR_DP) following a modified protocol of the Mao *et al.* [3] method. Prior to conjugation, the thiolated oligonucleotides were activated by the following procedure: 40 µL of 100 µM thiolated probe was mixed with 40 µL of 50 mM PB buffer (pH 8) and 4 µL of 100 mM dithiotreitol (DTT, Sigma Aldrich) to reduce the disulfide bond of thiol-modified oligonucleotides to the active sulfhydryl form. After incubation at room temperature for 60 min, excess DTT was removed by four extractions with 100 µL ethyl acetate solution.

Functionalization of AuNP was carried out by mixing 40 µL of freshly activated detector probe and 1 mL of AuNP solution. Then, 10 µL of 1% SDS and 20 µL of 0.5 mM PB (pH 8) were added, and the solution was incubated for 20 min at 23 °C with shaking on a thermomixer (600 rpm, Eppendorf Thermomixer Plus, Eppendorf, Hamburg, Germany). The solution was then subject to “aging” by the stepwise addition

of 2 M NaCl (every 30 min) until a final concentration of 0.4 M was reached. After overnight incubation at room temperature, unbound detector probes were removed by centrifugation (25 min, 12,000 rpm, 21 °C). The pellet was resuspended three times in 1 mL washing solution (0.1% Tween20) and, finally, in 1 mL ultrapure water. The resulting AuNP-DP conjugates were stored at 4 °C until preparation of the conjugate pad.

Gold-nanoparticles were characterized before and after functionalization by determining the maximum absorbance via photometrical screening from 400 to 800 nm (Perkin Elmer UV/VIS Spectrophotometer Lambda 16, Waltham, MA, USA). The results indicated a visible colour change and a slight shift in the maximum absorbance values from 519 to 525 nm (Figure S6).

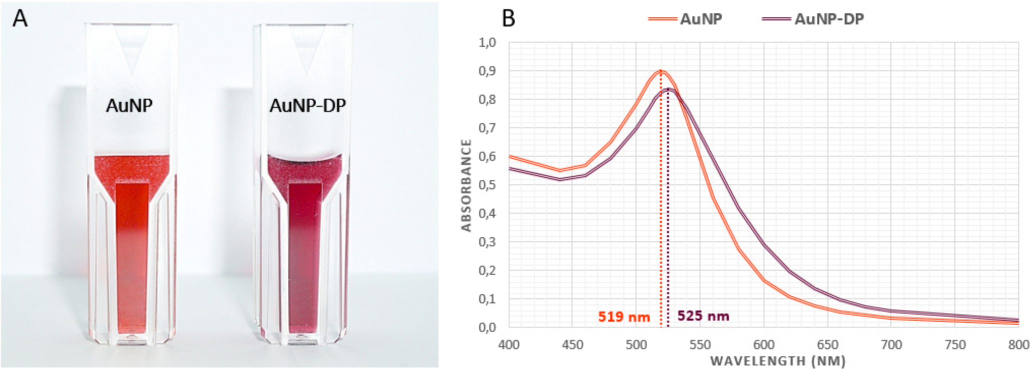

Supplementary Figure S6. Gold nanoparticles before and after functionalization with DNA detector probes. (A) Visible colour change from bright red (before functionalization) to dark red (after functionalization). (B) Photometric scan from 400 – 800 nm indicating a slight shift in the maximum absorbance values after functionalization (from 519 to 525 nm).

6. Preparation of streptavidin-biotin-probe conjugates (STV-Bio-CP and STV-Bio-CL)

Capture and control probes for the test and control lines, respectively, were deposited on the nitrocellulose membrane in the form of streptavidin-biotin-probe conjugates. To prepare these conjugates, 91.7 µL of 100 µM biotinylated probe (H-BacR_CP and H-CL, respectively) was mixed with 100 µL of 1 mg mL^{-1} streptavidin (Roth, Karlsruhe, Germany) and 200 µL of 50 mM PB buffer (pH 8). After incubation for one hour at room temperature, unbound DNA capture probes were removed by centrifugation for 10 min at 14,000 x g using a centrifugal filter (Roti®-Spin Mini-30, 30 kD cut-off, Roth, Karlsruhe, Germany). Both streptavidin-biotin-probe conjugates were recovered from the sample reservoir with 100 µL of 50 mM PB. Prior to immobilization on the nitrocellulose membrane, the STV-Bio-capture probe conjugate (for the test line) was diluted 1:10, and the STV-Bio-control probe conjugate (for the control line) was diluted 1:3 in 50 mM PB.

References

1 Turkevich, J., Stevenson, P. C. & Hillier, J. A study of the nucleation and growth processes in the synthesis of colloidal gold. *Discussions of the Faraday Society* **11**, 55-75, doi:10.1039/DF9511100055 (1951).

2 Frens, G. *Nature: Phys. Sci. 1973, 241, 20-22.(c) Hayat, MA Colloidal Gold: Principles, Methods and Applications*. (Academic Press: New York, 1989).

3 Mao, X. *et al.* Disposable Nucleic Acid Biosensors Based on Gold Nanoparticle Probes and Lateral Flow Strip. *Analytical Chemistry* **81**, 1660-1668, doi:10.1021/ac8024653 (2009).
